# Supplementary material for: Prognostic MicroRNA Panel for HCV-Associated HCC: Integrating Computational Biology and Clinical Validation
Source: Cancers (Basel). 2022 Jun 21;14(13):3036. doi: 10.3390/cancers14133036 (PMC9265118; doi:10.3390/cancers14133036)
Supplement: Supplementary file 1 [file cancers-14-03036-s001.zip › Table S1.pdf]

Table S1. DE-miRNAs from microarray dataset

miRNA ID	log fold change	Average expression	t	*P*-value	adjusted *P*-value	B statistics
**Upregulated**						
hsa-mir-1269	3.980556	3.006296	7.573041	3.48E-08	2.58E-05	8.862391
hsa-mir-224	2.268889	5.132963	4.317145	0.000186	0.00811	0.691497
hsa-mir-452	2.004444	5.007037	4.287352	0.000201	0.008498	0.615536
hsa-mir-130b	1.673889	8.126296	7.236449	8.09E-08	3.60E-05	8.064353
**Downregulated**						
hsa-mir-503	-1.56833	6.736667	-4.54318	0.000101	0.005233	1.270147
hsa-mir-424	-1.70222	6.841481	-5.2379	1.55E-05	0.00172	3.060813
hsa-mir-23a	-1.77944	4.24963	-4.71964	6.27E-05	0.004113	1.724066
hsa-miR-29b	-1.82667	5.335556	-4.69036	6.79E-05	0.004268	1.648638
hsa-mir-150	-1.82722	9.885926	-4.28367	0.000203	0.008498	0.60616
hsa-mir-139	-1.84667	5.137778	-4.99007	3.02E-05	0.002688	2.421497
hsa-mir-10a	-1.88222	7.034815	-4.77929	5.34E-05	0.003715	1.877774
hsa-mir-4269	-1.89722	4.128148	-7.62543	3.05E-08	2.58E-05	8.985176
hsa-mir-130a	-1.91	8.736667	-9.22381	6.86E-10	1.53E-06	12.5379
hsa-mir-27a	-1.98	3.592222	-4.42072	0.000141	0.00666	0.956184
hsa-mir-203	-2.28278	4.461852	-4.64132	7.75E-05	0.004542	1.522428
hsa-mir-886	-2.35333	7.171111	-5.02797	2.72E-05	0.002585	2.519308
hsa-mir-200c	-2.355	5.542222	-5.80061	3.41E-06	0.000632	4.504642
hsa-mir-214	-2.67	11.09444	-4.55014	9.92E-05	0.005233	1.288025
hsa-mir-214	-2.67667	5.091111	-7.08254	1.19E-07	4.43E-05	7.694318
hsa-mir-199b	-3.05111	10.51185	-5.73493	4.06E-06	0.000695	4.337026
hsa-miR-199a-3p	-3.22889	10.41593	-5.2558	1.47E-05	0.00172	3.106947
hsa-miR-199a-5p	-3.31389	10.00259	-5.88751	2.70E-06	0.000547	4.725912
