# Supplementary material for: Prognostic MicroRNA Panel for HCV-Associated HCC: Integrating Computational Biology and Clinical Validation
Source: Cancers (Basel). 2022 Jun 21;14(13):3036. doi: 10.3390/cancers14133036 (PMC9265118; doi:10.3390/cancers14133036)
Supplement: Supplementary file 1 [file cancers-14-03036-s001.zip › Table S2.pdf]

**Table S2. DE-miRNAs from TCGA dataset**

| miRNA ID | Log fold change | False discovery rate | Tumor | Normal | Delta |
|---|---|---|---|---|---|
| **Upregulated** | | | | | |
| hsa-mir-217 | 4.237359 | 0.00095 | 2742.886 | 145.6502 | 11622.59 |
| hsa-mir-224 | 3.02565 | 0.027267 | 349.2049 | 42.85636 | 1056.572 |
| hsa-mir-183 | 2.730561 | 0.048386 | 4087.799 | 617.5533 | 11161.99 |
| **Downregulated** | | | | | |
| hsa-mir-142 | -1.95755 | 0.011533 | 1599.147 | 6242.973 | 3130.404 |
| hsa-mir-199b | -1.72226 | 0.046202 | 946.2798 | 3129.33 | 1629.743 |
| hsa-mir-150 | -2.17145 | 0.002423 | 471.3316 | 2131.151 | 1023.473 |
| hsa-mir-424 | -2.72822 | 1.42E-05 | 140.6106 | 936.6654 | 383.6167 |
| hsa-mir-215 | -3.54143 | 4.24E-10 | 93.39001 | 1090.393 | 330.7346 |
| hsa-mir-3607 | -2.83414 | 7.02E-06 | 50.00577 | 358.5205 | 141.7232 |
