# Supplementary material for: Prognostic MicroRNA Panel for HCV-Associated HCC: Integrating Computational Biology and Clinical Validation
Source: Cancers (Basel). 2022 Jun 21;14(13):3036. doi: 10.3390/cancers14133036 (PMC9265118; doi:10.3390/cancers14133036)
Supplement: Supplementary file 1 [file cancers-14-03036-s001.zip › Table S3.pdf]

Table S3. Correlation between the targe miRNAs in HCV cirrhotic and HCV-HCC groups and with other clinicopathological factors.

Factor		miR-142 $2^{-\Delta\Delta Ct}$	miR-150 $2^{-\Delta\Delta Ct}$	miR-424 $2^{-\Delta\Delta Ct}$	miR-3607 $2^{-\Delta\Delta Ct}$	miR-215 $2^{-\Delta\Delta Ct}$	miR-183 $2^{-\Delta\Delta Ct}$	miR-199a $2^{-\Delta\Delta Ct}$	miR-224 $2^{-\Delta\Delta Ct}$	miR-217 $2^{-\Delta\Delta Ct}$
**miR-142 $2^{-\Delta\Delta Ct}$**	**Correlation**	1.000								
	**Significance (2-tailed)**									
	**df**	0								
**miR-150 $2^{-\Delta\Delta Ct}$**	**Correlation**	0.197	1.000							
	**Significance (2-tailed)**	0.028*								
	**df**	122	0							
**miR-424 $2^{-\Delta\Delta Ct}$**	**Correlation**	0.156	0.544	1.000						
	**Significance (2-tailed)**	0.084	0.000**							
	**df**	122	122	0						
**miR-3607 $2^{-\Delta\Delta Ct}$**	**Correlation**	-0.012	0.149	0.168	1.000					
	**Significance (2-tailed)**	0.898	0.098	0.062						
	**df**	122	122	122	0					
**miR-215 $2^{-\Delta\Delta Ct}$**	**Correlation**	-0.065	0.053	0.120	0.170	1.000				
	**Significance (2-tailed)**	0.471	0.562	0.183	0.060					
	**df**	122	122	122	122	0				
	**Correlation**	-0.058	0.099	0.364	0.218	0.361	1.000			

**miR-183 $2^{-\Delta\Delta Ct}$**	**Significance (2-tailed)**	0.524	0.274	0.000**	0.015	0.000**				
	**df**	122	122	122	122	122	0			
**miR-199a $2^{-\Delta\Delta Ct}$**	**Correlation**	0.223	0.386	0.555	0.089	0.246	0.271	1.000		
	**Significance (2-tailed)**	0.013*	0.000**	0.000**	0.325	0.006**	0.002**			
	**df**	122	122	122	122	122	122	0		
**miR-224 $2^{-\Delta\Delta Ct}$**	**Correlation**	0.103	0.461	0.359	-0.013	0.002	0.052	0.515	1.000	
	**Significance (2-tailed)**	0.257	0.000**	0.000**	0.890	0.985	0.569	0.000**		
	**df**	122	122	122	122	122	122	122	0	
**miR-217 $2^{-\Delta\Delta Ct}$ e**	**Correlation**	-0.035	0.063	0.098	0.296	0.529	0.289	0.198	-0.046	1.000
	**Significance (2-tailed)**	0.700	0.488	0.277	0.001**	0.000**	0.001**	0.028*	0.612	
	**df**	122	122	122	122	122	122	122	122	0
**Age**	**Correlation**	0.095	-0.021	0.069	-0.095	0.167	0.071	0.075	-0.020	0.023
	**Significance (2-tailed)**	0.296	0.814	0.447	0.296	0.063	0.431	0.410	0.829	0.802
	**df**	122	122	122	122	122	122	122	122	122
**Gender**	**Correlation**	0.024	0.012	0.095	-0.005	0.011	0.133	0.160	0.006	0.012
	**Significance (2-tailed)**	0.791	0.894	0.295	0.960	0.903	0.142	0.075	0.946	0.896
	**df**	122	122	122	122	122	122	122	122	122
**ALT**	**Correlation**	-0.094	-0.019	-0.093	-0.166	-0.103	-0.117	-0.050	0.034	-0.091
	**Significance (2-tailed)**	0.297	0.834	0.305	0.065	0.253	0.196	0.582	0.710	0.317
	**df**	122	122	122	122	122	122	122	122	122
**AST**	**Correlation**	-0.097	0.017	-0.045	-0.133	-0.092	-0.106	-0.025	0.134	-0.033
	**Significance (2-tailed)**	0.282	0.855	0.623	0.140	0.309	0.242	0.780	0.136	0.717

	**df**	122	122	122	122	122	122	122	122	122
**AFP**	**Correlation**	-0.129	-0.149	-0.153	-0.043	-0.065	-0.159	-0.105	-0.144	-0.069
	**Significance (2-tailed)**	0.154	0.098	0.089	0.638	0.470	0.078	0.247	0.111	0.443
	**df**	122	122	122	122	122	122	122	122	122
**T. Bil**	**Correlation**	0.032	-0.034	0.050	0.008	0.044	0.091	0.045	0.068	-0.002
	**Significance (2-tailed)**	0.723	0.707	0.578	0.930	0.627	0.315	0.619	0.456	0.981
	**df**	122	122	122	122	122	122	122	122	122
**D. Bil**	**Correlation**	0.021	-0.027	0.067	0.016	0.049	0.102	0.031	0.057	0.005
	**Significance (2-tailed)**	0.814	0.768	0.458	0.857	0.587	0.260	0.729	0.529	0.954
	**df**	122	122	122	122	122	122	122	122	122
**Albumin**	**Correlation**	0.123	0.125	0.025	0.015	-0.004	-0.154	-0.027	-0.090	0.035
	**Significance (2-tailed)**	0.172	0.167	0.781	0.869	0.966	0.089	0.766	0.320	0.703
	**df**	122	122	122	122	122	122	122	122	122

Association was determined using Spearman's correlation. Rho: Spearman's rank correlation coefficient. * Correlation is significant as P-value ≤ 0.05 (two-tailed), ** Correlation is significant as P-value ≤ 0.01 (two-tailed).
